# Characterization of Clinically-Attenuated *Burkholderia mallei* by Whole Genome Sequencing: Candidate Strain for Exclusion from Select Agent Lists

**DOI:** 10.1371/journal.pone.0002058

**Published:** 2008-04-30

**Authors:** Steven E. Schutzer, Linda R. K. Schlater, Catherine M. Ronning, David DeShazer, Benjamin J. Luft, John J. Dunn, Jacques Ravel, Claire M. Fraser-Liggett, William C. Nierman

**Affiliations:** 1 Department of Medicine, University of Medicine and Dentistry - New Jersey Medical School, Newark, New Jersey, United States of America; 2 United States Department of Agriculture, Ames, Iowa, United States of America; 3 J. Craig Venter Institute, Rockville, Maryland, United States of America; 4 U.S. Army Medical Research Institute of Infectious Diseases, Fort Detrick, Maryland, United States of America; 5 Department of Medicine, State University of New York, Stony Brook, New York, United States of America; 6 Biology Department, Brookhaven National Laboratory, Upton, New York, United States of America; 7 Institute for Genome Sciences, Department of Medicine, University of Maryland School of Medicine, Baltimore, Maryland, United States of America; 8 Institute for Genome Sciences, Department of Microbiology and Immunology, University of Maryland School of Medicine, Baltimore, Maryland, United States of America; 9 Department of Biochemistry and Molecular Biology, The George Washington University School of Medicine, Washington, D. C., United States of America; Massachusetts General Hospital, United States of America

## Abstract

**Background:**

*Burkholderia mallei* is an understudied biothreat agent responsible for glanders which can be lethal in humans and animals. Research with this pathogen has been hampered in part by constraints of Select Agent regulations for safety reasons. Whole genomic sequencing (WGS) is an apt approach to characterize newly discovered or poorly understood microbial pathogens.

**Methodology/Principal Findings:**

We performed WGS on a strain of *B. mallei*, SAVP1, previously pathogenic, that was experimentally infected in 6 equids (4 ponies, 1 mule, 1 donkey), natural hosts, for purposes of producing antibodies. Multiple high inocula were used in some cases. Unexpectedly SAVP1 appeared to be avirulent in the ponies and mule, and attenuated in the donkey, but induced antibodies. We determined the genome sequence of SAVP1 and compared it to a strain that was virulent in horses and a human. In comparison, this phenotypic avirulent SAVP1 strain was missing multiple genes including all the animal type III secretory system (T3SS) complex of genes demonstrated to be essential for virulence in mice and hamster models. The loss of these genes in the SAVP1 strain appears to be the consequence of a multiple gene deletion across insertion sequence (IS) elements in the *B. mallei* genome. Therefore, the strain by itself is unlikely to revert naturally to its virulent phenotype. There were other genes present in one strain and not the other and vice-versa.

**Conclusion/Significance:**

The discovery that this strain of *B. mallei* was both avirulent in the natural host ponies, and did not possess T3SS associated genes may be fortuitous to advance biodefense research. The deleted virulence-essential T3SS is not likely to be re-acquired naturally. These findings may provide a basis for exclusion of SAVP1 from the Select Agent regulation or at least discussion of what else would be required for exclusion. This exclusion could accelerate research by investigators not possessing BSL-3 facilities and facilitate the production of reagents such as antibodies without the restraints of Select Agent regulation.

## Introduction

An initial approach today to a newly discovered pathogen is to perform whole genome sequencing (WGS). This same approach is relevant for investigations of an understudied high-consequence pathogen such as *Burkholderia mallei*, the cause of glanders. This zoonotic bacterium can kill humans and animals [1], [2]. It is classified as a Category B biothreat agent and it has been used as a biological warfare agent [2]–[8]. Its ease of use against civilians and lack of countermeasures has compelled several agencies to rank it very high on their priority lists of biothreat agents[9], [10]. Its ease of use as a biothreat agent is illustrated by the physician who, even in 1915, grew it out of his Washington DC area home, and distributed it for an attack against US horses destined for Europe as critical transportation in World War I[11]. Humans were also subject to infection. We remain highly vulnerable to this agent because there is no rapid diagnostic assay, no distinctive diagnostic signs, and the incubation period is short, a few days. In addition there is no vaccine, no infection-induced immunity, and limited reliable *in vivo* data on antibiotic efficacy. Overall, our general knowledge of this understudied pathogen and its disease is limited [2], [12]–[17]. Our desire to decrease our vulnerabilities to this pathogen, as part of the national biodefense efforts, is hampered, in part, by constraints of the Select Agent regulation and the need for BSL-3 facilities. These are appropriately in place for safety reasons, however, the availability of a suitable attenuated surrogate strain would be desirable as it could accelerate *B. mallei* related biodefense research in many non-BSL-3 laboratories.

*B. mallei* [18] is a Gram-negative non-motile aerobic bacteria with a genome of approximately 6 Mb organized in two circular chromosomes. In addition to infecting humans, *B. mallei* can cause acute or chronic fatal contagious zoonotic infections in its natural equine host, such as horses, donkeys, and mules, with a very low infectious dose. Two major potential routes of infection for a biologic attack are aerosol and cutaneous contact. Gastrointestinal ingestion is a common mode of natural infection in equines. The incubation period is typically between 3–6 days but may be longer.

An example of a phenotypic highly virulent strain is *B. mallei* strain ATCC 23344 (China 7). It is highly virulent in its natural hosts, equines, in humans, and in mice and hamster models[14], [19]–[21]. This strain of *B. mallei* contains an animal type III secretion system (T3SS) gene complex which is essential for virulence[15], [22]. Genome sequencing (WGS) and analysis of this strain identified a number of other putative virulence factors whose function was supported by comparative genome hybridization and expression profiling of the bacterium in hamster liver *in vivo* [22]. Numerous insertion sequence elements that have mediated extensive deletions and rearrangements of the genome relative to the *B. pseudomallei* genome were found. As part of our interest to study mechanisms responsible for virulence we performed WGS and comparative genomic analysis on strains of *B. mallei* in which we had closely linked information from an actual clinical infection in its natural host or a suitable animal model rather than using an archived strain lacking this information. We intended to compare the gene content of phenotypic virulent to avirulent strains to identify candidate virulence genes. One serendipitous event and an unexpected finding led to what may be a fortuitous observation. The serendipitous event occurred when five of six equids were infected, but not made ill, with what was a previously pathogenic strain of *B. mallei*, designated as SAVP1. At the time of inoculation this strain was still believed to be pathogenic. A sixth equid, a donkey, developed clinical signs only after a massive exposure. The strain appeared avirulent even when administered in escalating doses. The other unexpected finding came while performing comparative genomic analysis between SAVP1 and the strain of *B. mallei* that was currently behaving as virulent in humans and horses. This finding is both informational and fortuitous because it may open the door for SAVP1 to be classified as suitably avirulent for exclusion from Select Agent lists[23] thereby expanding our biodefense research efforts.

## Results

### In vivo characterization of phenotypic avirulent strain of B. mallei in natural equine hosts

Based upon our premise that relevant pathogenic differences between strains may be revealed by WGS of a bona-fide phenotypic avirulent strain of *B. mallei,* we wanted to select a strain that was previously evaluated in the natural host. This was accomplished by selecting a strain (SAVP1) that had previously caused disease in a mule (in India) and surprisingly did not produce overt disease when inoculated, first orally and subsequently intravenously, into a mule and 4 ponies, even at escalating doses approaching 10^9^ colony forming units (CFU) per ml. The prime purpose of the initial experiment, carried out by one of us (LKS) more than twenty years ago, was to develop a diagnostic assay for *B. mallei* as part of the US Department of Agricultures mission to help prevent the disease from entering the United States through horses. It was not meant as a controlled infectivity study, consequently full clinical description and detailed history of the strain are not available. Notes indicate 4 equids (4 ponies, a mule, and a donkey) were exposed by pharyngeal spray twice to increasing doses of *B. mallei*. When none of the equids became ill or seroconverted by 30 days post exposure, they were challenged intravenously with 5 ml of heavy suspension of organisms. The extreme exposure produced clinical signs only in the donkey, a species known to be more susceptible than horses and mules to *B. mallei*. The donkey was euthanized for humane reasons. This donkey received a high intravenous dose, approaching believed to have approached 5 ml of 10^9^ cfu/ml – thus the symptoms may have been related to endotoxin. Clinical signs in the ponies and the mule were either minimal (fever) or nonexistent. All 6 equids produced antibodies to *B mallei* as a result of the intravenous exposure. The expected clinical signs of morbid infection were not present in the ponies or the mule. At the time of the necropsy, there was little evidence of systemic pathology. This included the absence of the common sinopulmonary findings of purulent nasal discharge, ulcerations, pleural effusion, pulmonary edema, congestion, or pneumonia. The sole pulmonary involvement was restricted to a few minimal bronchopulmonary granulomatous lesions which did not grow *B. mallei* on culture. From a microbiologic vantage it was not possible to ascertain if current avirulence was an attenuation effect that arose from passage in the mule before it was grown in culture and re-inoculated or if it was an effect of the *in vitro* culture. What is important here is the observation that this particular strain had avirulent/attenuated behavior in this natural host experiment. We performed WGS on DNA extracted from the same or near (low-passage) generation growth of the *B. mallei* used in this experiment.

### Detection of genes present or absent in an avirulent strain from a natural equine host in comparison to a known virulent strain

**Figure 1:**
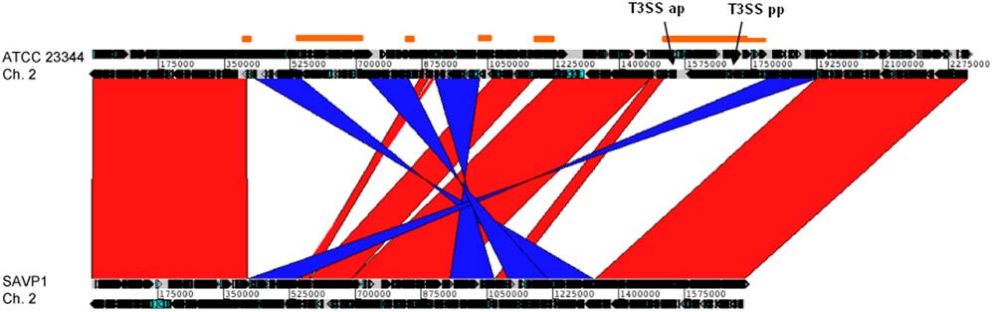
Comparison of the small chromosomes of *B. mallei* ATCC 23344 (top) and *B. mallei* SAVP1 (bottom) using ACT and the whole genome alignment MUMmer. Regions of similarity, rearrangements, and deletions are readily apparent between these two strains. The red and blue bands represent the forward and reverse matches, respectively. The orange horizontal bars represent the regions of ATCC 23344 that are missing in SAVP1. The locations of the animal pathogen-like T3SS (T3SS ap) and the plant pathogen-like (T3SS pp) gene clusters that are present in ATCC 23344, but absent in SAVP1, are indicated by arrows. The ATCC 23344 small chromosome (CP000011) is 2.32 Mb and the SAVP1 small chromosome (CP000525) is 1.73 Mb.

The SAVP1 strain was subjected to WGS and the resulting data compared those from the virulent ATCC 23344 strain[22] which was know to have caused recent near-death in a human and five horses that had to be euthanized[19], [21]. A one way analysis interrogated what genes were present in ATCC 23344 but lacking in the SAVP1 strain. This analysis took all of the ATCC 23344 coding sequences (CDS) and aligned them against the SAVP1 genomic sequence, identifying 582 ORFs that were present in ATCC 23344 but not SAVP1, the vast majority of which exist within a few large gene clusters (See Table 1, Supplementary Table S1, and Figure 1). In contrast, all of the genes shared between the two isolates are at least 99% identical over 99% or more of their length, as determined by blastn.

**Table 1:** Type III secretion proteins encoded by genes present in ATCC 23344 but absent in SAVP1.

Locus	Annotation	5′ end	3′ end
BMA_A1520	type III secretion chaperone BicP	1650045	1649590
BMA_A1532	type III secretion chaperone BicA	1662375	1661830
BMA_A1533	type III secretion system protein BsaZ	1663706	1662471
BMA_A1534	type III secretion system protein BsaY	1664480	1663710
BMA_A1535	type III secretion system protein BsaX	1664753	1664499
BMA_A1536	type III secretion system protein BsaW	1665469	1664789
BMA_A1537	type III secretion system protein BsaV	1666442	1665459
BMA_A1540	type III secretion system protein BsaS	1669435	1668125
BMA_A1541	type III secretion system protein BsaR	1669839	1669432
BMA_A1542	type III secretion system protein BsaQ	1671923	1669851
BMA_A1543	type III secretion system protein BsaP	1673080	1671959
BMA_A1544	type III secretion system protein BsaO	1674897	1673077
BMA_A1545	type III secretion system transcriptional regulator BsaN	1675726	1674968
BMA_A1547	type III secretion system protein BsaM	1676120	1677406
BMA_A1548	type III secretion system protein BsaL	1677403	1677672
BMA_A1550	type III secretion system BasJ	1678034	1678984
BMA_A1551	type III secretion apparatus protein OrgA/MxiK	1678981	1679568
BMA_A1552	type III secretion apparatus protein, HrpE/YscL family	1679537	1680331
BMA_A1602	type III secretion outer membrane pore, YscC/HrcC family	1740918	1739119
BMA_A1613	type II/III secretion system family protein	1754093	1752303
BMA_A1625	type III secretion inner membrane protein, authentic frameshift	1763834	1762891
BMA_A1627	type III secretion inner membrane protein SctS	1765159	1764896
BMA_A1628	type III secretion inner membrane protein SctR	1765850	1765200
BMA_A1629	type III secretion inner membrane protein SctQ	1767135	1765837
BMA_A1630	type III secretion inner membrane protein SctV	1769843	1767771
BMA_A1631	type III secretion protein, YscU/HrpY family	1770922	1769840
BMA_A1632	type III secretion protein, HrpB1/HrpK family	1771158	1771733
BMA_A1633	type III secretion protein HrpB2	1771747	1772163
BMA_A1634	type III secretion inner membrane protein SctJ, authentic frameshift	1772166	1773012
BMA_A1635	type III secretion protein HrpB4	1773009	1773686
BMA_A1636	type III secretion inner membrane protein SctL	1773671	1774387
BMA_A1637	type III secretion apparatus H+-transporting two-sector ATPase	1774423	1775712

All genes are located on chromosome II and appear to be present in two basically contiguous segments: BMA_A1520-BMA_A1552 and BMA_A1625-BMA_A1637. Genes with Bsa are part of the animal pathogen-like T3SS. A complete list of the genes present in ATCC 23344 but missing from SAVP1 is given in Supplementary Table S1.

**Figure 2:**
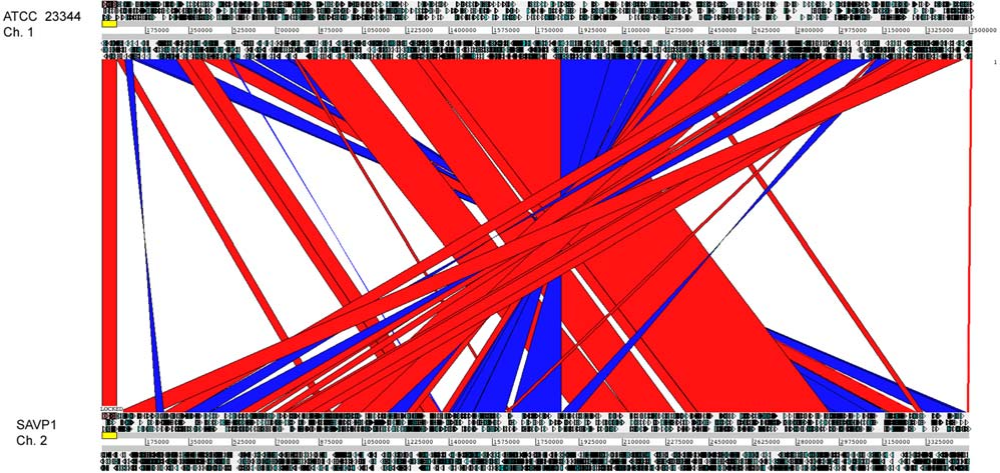
Comparison of the large chromosomes of *B. mallei* ATCC 23344 (top) and *B. mallei* SAVP1 (bottom) using ACT and the whole genome alignment MUMmer. Regions of similarity, rearrangements, and deletions are readily apparent between these two strains. The red and blue bands represent the forward and reverse matches, respectively. The ATCC 23344 large chromosome (CP000010) is 3.51 Mb and the SAVP1 large chromosome (CP000526) is 3.49 Mb.

The most noteworthy difference was the loss of all the animal T3SS associated genes[15] in an apparent IS mediated deletion containing contiguous genes (two sets of T3SS: one consists of 23 genes from BMA_A1530-A1552 (the Bsa genes) and a second set from BMA_A1625-A1637 consists of 13 genes, Genbank accession numbers, ATCC 23344 small chromosome- CP000011 and SAVP1- CP000525). These genes are located within the largest of the ATCC 23344-specific gene clusters mentioned above. The absence of animal type T3SS alone could explain its avirulence in the natural host. Differences in the ATCC 23344 and SAVP1 genomes result from multiple inversions and deletions of genome segments at insertion sequences (IS), particularly IS407A elements (Fig. 1 and 2). SAVP1 has lost approximately 610 kb of DNA due to IS mediated deletions (see Supplemental Table 1 for genes not present in SAVP1). Most of the lost genes are on chromosome 2, a replicon that encodes more accessory functions than chromosome 1. Notable in comparing presence of genes on SAVP1 but not on ATCC 23344 include multiple fimbriae/pili genes and several hypothetical genes (See Figure 2). The rearrangements detailed here demonstrate the plasticity of the *B. mallei* genome and suggest that IS mediated deletions may have a profound effect on the relative virulence of *B. mallei* strains.

Thus, based on our current knowledge, the strain is unlikely to undergo a natural restoration to a virulent state by passage in an animal host, in contrast to the situation if there were only reparable replication errors or point mutations in a single or a few genes.

## Discussion

The general public remains at risk to the pathogenic effects of *B. mallei* until we can develop reliable therapeutics, vaccines or other protective measures, and rapid diagnostics. We would have an opportunity to develop these countermeasures more quickly if we could identity attenuated strains of *B. mallei* and employ the strains in selected experiments. Unlike anthrax, where we have effective diagnostics, approved therapy, and vaccines, none of these resources are available for *B. mallei*. This means that those most capable of advancing research in this field are at high occupational risk, without effective contingency measures, should an infection be suspected or actually occur. In addition to occupational risk, two other factors that impede research are the requirement to use BSL-3 facilities and the Select Agent regulations[23], [24]. Fortunately, there are provisions within the Select Agent Program that enable a particular microbial strain to removed or excluded[25]. Certain strains of microbes have already been excluded from the list and not subject to the requirements of 42 CFR Part 73 and 9 CFR Part 121 if used in basic or applied research, as positive controls, for diagnostic assay development, proficiency testing, or for the development of vaccines and therapeutics. Examples now excluded are certain strains of *Yersinia pestis, Bacillus anthracis* strains devoid of both plasmids pX01 and pX02, *Bacillus anthracis* strains devoid of the plasmid pX02 (e.g., *Bacillus anthracis* Sterne, pX01+pX02-), *Brucella abortus* Strain 19 and strain RB51 (vaccine strains), *Coxiella burnetii, Francisella tularensis* subspecies *novicida*, and *Francisella tularensis* subspecies *holartica* LVS (live vaccine strain)[25]. However, the regulations go back into effect if there is any reintroduction of factor(s) associated with virulence or other manipulations that restore the virulence or diminish the attenuation. Therefore, as might be done with excluded strains above, we believe it is prudent to monitor for any possible reversions with the SAVP1 during *in vivo* experiments. The same caveat applies to restoration experimentation, if it could be done, to provide more definitive evidence of that absence of T3SS as necessary and sufficient for attentuation.

We believe it is preferable to pair the strain with clinical cases in a natural-host infection whether that occurs naturally or by experiment. The closer the strain isolate is to the case the better, rather than an isolate from multiple *in vitro* passages in cultures. Misleading data might result by randomly selecting an isolate from a strain collection without the associated clinical information. In the current example we happened to identify the loss of animal T3SS genes in a strain associated with avirulent phenotypic behavior in its natural host. Because the T3SS is so essential to export of virulent factors, and this was unexpectedly found by WGS of SAVP1, there is increased confidence that experiments with this isolate may be accomplished with a higher degree of safety that with the virulent strain. Loss of some of the other 582 ORFs could be additive to this effect. Presence of other genes which are on SAVP1 and not on ATCC 23344 could also contribute to attenuated behavior. Though this strain would no longer be our choice for comparative genomic studies to detect subtle genetic differences that may account for virulence, its ability to evoke antibody responses confers it with great potential for other types of biodefense research as well as potential vaccines in equids and humans. Other avirulent strains may also prove to be candidates for Select Agent regulation exclusion[16], [17].

In summary, our investigation into virulence factors using WGS on clinically-associated strains of *B. mallei* led to an unexpected finding. This finding may serve to eventually have at least one strain, SAVP1, removed from select agent constraints. Its research utility could be assessed. We believe there are comparable situations with other biothreat agents. We hope that the example of our finding with SAVP1 will engender discussion among public health and regulatory agencies, academia, and the private sector that will favorably impact our biodefense research efforts.

## Materials and Methods

### Sequencing

The genomes of *B. mallei* were sequenced and assembled by random shotgun method as described[22].

### Coding Sequence (CDS) Prediction and Gene Identification

Open reading frames (ORFs) likely to encode proteins (CDSs) were identified by using GLIMMER. Identified CDSs were annotated by manual curation of the outputs of a variety of similarity searches. Searches of the predicted coding regions were performed with BLASTP, as described[26]. The protein–protein matches were aligned with blast_extend_repraze, a modified Smith-Waterman[27] algorithm that maximally extends regions of similarity across frameshifts. Gene identification is facilitated by searching against a database of nonredundant bacterial proteins (nraa) developed at The Institute for Genomic Research (TIGR) and curated from the public archives GenBank, Genpept, Protein Information Resource, and SwissProt. Searches matching entries in nraa have the corresponding role, gene common name, percent identity and similarity of match, pairwise sequence alignment, and taxonomy associated with the match assigned to the predicted coding region and stored in the database. CDSs were also analyzed with two sets of hidden Markov models constructed for a number of conserved protein families from PFAM and TIGRFAM. Regions of the genome without CDSs and CDSs without a database match were reevaluated by using BLASTX as the initial search, and CDSs were extrapolated from regions of alignment. Finally, each putatively identified gene was assigned to one of 113 role categories.

### ATCC 23344 - SAVP1 Comparative Analysis

All CDSs from ATCC 23344 were aligned against the whole genome sequence of SAVP1 with the Program to Assemble Spliced Alignments (PASA) [28]. PASA first summons BLAT[29] to align the CDSs to the genome and then validates each alignment by requiring a minimum 95% sequence identity over at least 90% of the gene length. Alignments failing BLAT validation are then realigned using sim4[30] and revalidated using the same criteria. All ATCC 23344 CDSs that could not be aligned were thus assumed to be absent from SAVP1. Similar analyses were applied to reverse strain comparisons.

### Animal Studies

The experimental infection of equids was performed under the auspices and regulations of the United States Department of Agriculture on Plum Island.

## Supporting Information

Table S1 Table of Genes present in ATCC23344 but absent from SAVP1
(0.08 MB PDF)
