# Supplementary material for: Characterization of Clinically-Attenuated Burkholderia mallei by Whole Genome Sequencing: Candidate Strain for Exclusion from Select Agent Lists
Source: PLoS One. 2008 Apr 30;3(4):e2058. doi: 10.1371/journal.pone.0002058 (PMC2312325; doi:10.1371/journal.pone.0002058)
Supplement: Table S1 — Table of Genes present in ATCC23344 but absent from SAVP1 (0.08 MB PDF) [file pone.0002058.s001.pdf]

Supplementary Table 1. Genes present in ATCC 23344 but absent from SAVP1. Gene clusters are separated by horizontal lines. T3SS genes are in bold. Chr = chromosome

Chr	Locus	Annotation	5' end	3' end	Role
I	BMA_0575	conserved hypothetical protein	601822	600776	Hypothetical proteins
I	BMA_0576	YdjC family protein	602706	601819	Unknown function
I	BMA_0577	radical SAM domain protein	604155	602710	Unknown function
I	BMA_0578	glycosyl transferase, group 2 family protein	605421	604243	Cell envelope
I	BMA_0579	conserved domain protein, degenerate	605741	605418	Hypothetical proteins
I	BMA_0580	luciferase-like monooxygenase	606048	607106	Unknown function
I	BMA_0581	hydrolase, alpha/beta fold family	608570	607542	Unknown function
I	BMA_0582	pyridoxal kinase	609093	609956	Biosynthesis of cofactors, prosthetic groups, carriers
I	BMA_0582.1	hypothetical protein	610426	610166	
I	BMA_0584	non-hemolytic phospholipase C	610626	612728	Cellular processes; Fatty acid and phospholipid metabolism
I	BMA_0756	phage integrase, truncation	787867	788043	Mobile and extrachromosomal elements
I	BMA_0950	transcriptional regulator, MerR family	1000883	1000692	Regulatory functions
I	BMA_0951	hypothetical protein	1001318	1000929	
I	BMA_0952	efflux transporter, outer membrane factor (OMF) lipoprotein, NodT family	1003263	1001359	Cellular processes; Transport and binding proteins
I	BMA_0953	AcrB/AcrD/AcrF family protein	1006436	1003266	Cellular processes; Transport and binding proteins
I	BMA_0954	efflux transporter, RND family, MFP subunit	1007548	1006433	Transport and binding proteins
I	BMA_0955	transcriptional regulator, TetR family	1007757	1008377	Regulatory functions
I	BMA_0956	chloride transporter, chloride channel (ClC) family	1010409	1008670	Transport and binding proteins
I	BMA_0957	putative membrane protein	1011544	1010519	Cell envelope
I	BMA_0958	transcriptional regulator, LysR family	1011700	1012593	Regulatory functions
I	BMA_0959	hypothetical protein	1013140	1012655	
I	BMA_0960	AP endonuclease, family 2	1013325	1014089	DNA metabolism
I	BMA_0961	2-dehydro-3-deoxygluconokinase	1014173	1015162	Energy metabolism
I	BMA_0962	transporter, major facilitator family	1015247	1016524	Transport and binding proteins
I	BMA_0963	2-ketogluconate reductase	1016529	1017506	Energy metabolism
I	BMA_0964	hypothetical protein	1017529	1018107	
I	BMA_0965	transcriptional regulator, lacI family	1018104	1019147	Regulatory functions
I	BMA_0966	putative membrane protein	1019205	1019993	Cell envelope
I	BMA_0967	hypothetical protein	1019966	1020370	
I	BMA_0968	conserved hypothetical protein	1021185	1020736	Hypothetical proteins
I	BMA_0969	polyhydroxybutyrate depolymerase	1021361	1022386	Energy metabolism; Fatty acid and phospholipid metabolism
I	BMA_0970	conserved hypothetical protein	1023339	1022824	Hypothetical proteins

I	BMA_0971	putative nonribosomal peptide synthetase, degenerate	1023457	1024878	Cellular processes
I	BMA_0971.1	hypothetical protein	1025144	1024869	
I	BMA_0972	transcriptional regulator, AsnC family	1025263	1025724	Regulatory functions
I	BMA_0973	conserved hypothetical protein	1025779	1026861	Hypothetical proteins
I	BMA_0974	hypothetical protein	1027214	1026891	
I	BMA_0975	hypothetical protein	1027582	1027211	
I	BMA_0976	saccharopine dehydrogenase	1027674	1028774	Amino acid biosynthesis
I	BMA_0977	conserved hypothetical protein	1029597	1028977	Hypothetical proteins
I	BMA_0978	cyd operon protein YbgT	1029755	1029594	Energy metabolism
I	BMA_0979	magnesium and cobalt transport protein CorA	1029960	1030937	Transport and binding proteins
I	BMA_0980	conserved hypothetical protein	1031235	1030945	Hypothetical proteins
I	BMA_0983	putative lipoprotein	1031787	1032227	Cell envelope
I	BMA_0984	hypothetical protein	1033103	1032876	
I	BMA_0985	hypothetical protein	1033669	1033941	
I	BMA_0986	transporter, small conductance mechanosensitive ion channel (MscS) family	1036373	1033947	Transport and binding proteins
I	BMA_0987	regulatory protein NosR	1038869	1036737	Regulatory functions
I	BMA_0988	cytochrome c family protein	1039422	1038928	Energy metabolism
I	BMA_0989	hypothetical protein	1039848	1039444	
I	BMA_0990	hypothetical protein	1040120	1039851	
I	BMA_0991	putative protein disulfide isomerase NosL	1040661	1040131	Central intermediary metabolism; Protein fate
I	BMA_0992	copper ABC transporter, permease protein	1041494	1040658	Transport and binding proteins
I	BMA_0993	copper ABC transporter, ATP-binding protein	1042420	1041491	Transport and binding proteins
I	BMA_0994	copper ABC transporter, periplasmic copper-binding protein	1043652	1042402	Transport and binding proteins
I	BMA_0995	nitrous-oxide reductase	1045792	1043825	Energy metabolism
I	BMA_0996	ApbE family protein	1046443	1047501	Biosynthesis of cofactors, prosthetic groups, carriers
I	BMA_0998	putative drug resistance ABC-2 type transporter, permease protein	1048524	1049264	Cellular processes; Transport and binding proteins
I	BMA_0999	methyl-accepting chemotaxis protein	1049535	1051028	Cellular processes
I	BMA_0999.1	conserved hypothetical protein	1051223	1051633	Hypothetical proteins
I	BMA_1001	putative dihydroxyacetone kinase	1051849	1053561	Energy metabolism
I	BMA_1002	conserved domain protein	1054985	1053936	Hypothetical proteins
I	BMA_1003	conserved domain protein	1055983	1054985	Hypothetical proteins
I	BMA_1004	transporter, monovalent cation:proton antiporter-2 (CPA2) family	1057324	1055990	Transport and binding proteins
I	BMA_1005	putative tryptophan halogenase	1058852	1057410	Energy metabolism
I	BMA_1006	hypothetical protein	1059586	1058990	
I	BMA_1007	hypothetical protein	1059735	1060253	
I	BMA_1009	NAD-dependent methanol dehydrogenase, authentic frameshift	1060644	1061815	Energy metabolism

I	BMA_1010	hypothetical protein	1062317	1061787	
I	BMA_1011	conserved hypothetical protein	1062435	1063196	Hypothetical proteins
I	BMA_1012	esterase, PHB depolymerase family	1064587	1063499	Fatty acid and phospholipid metabolism
I	BMA_1014	hypothetical protein	1064915	1065181	
I	BMA_1015	conserved hypothetical protein	1065172	1065639	Hypothetical proteins
I	BMA_1021	type-1 fimbrial protein, authentic frameshift	1068107	1068636	Cell envelope
I	BMA_1022	fimbrial chaperone protein	1068735	1069505	Cell envelope
I	BMA_1023	fimbrial usher protein	1069512	1072064	Cell envelope; Protein fate
I	BMA_1024	type-1 fimbrial protein, A subunit	1072412	1073026	Cell envelope
I	BMA_1025	hypothetical protein	1073653	1073192	
I	BMA_1026	conserved hypothetical protein	1073869	1074054	Hypothetical proteins
I	BMA_1027	haemagglutinin family protein	1074198	1077236	Cellular processes
I	BMA_1027.1	hypothetical protein	1078463	1079077	
I	BMA_1034	DNA-binding response regulator MrgR	1079375	1080040	Cellular processes; Regulatory functions; Signal transduction
I	BMA_1761	osmotically inducible lipoprotein B	1850462	1850674	Cellular processes
I	BMA_1762	putative lipoprotein	1850926	1851150	Cell envelope
I	BMA_2089	putative outer membrane porin	2181522	2182661	Transport and binding proteins
I	BMA_2689	putative addiction module killer protein	2788382	2788678	Mobile and extrachromosomal elements
I	BMA_2690	putative addiction module antidote protein	2788681	2789037	Mobile and extrachromosomal elements
I	BMA_2791	cold-shock domain family protein	2881742	2881539	Cellular processes
II	BMA_A0416	methyltransferase domain protein	411529	410549	Unknown function
II	BMA_A0417	permease, cytosine/purines, uracil, thiamine, allantoin family	412046	413470	Transport and binding proteins
II	BMA_A0418	NAD-dependent deacetylase, Sir2 family	413467	414405	Regulatory functions
II	BMA_A0419	conserved hypothetical protein	414431	415045	Hypothetical proteins
II	BMA_A0420	6-phosphogluconate dehydrogenase, decarboxylating	415501	416916	Energy metabolism
II	BMA_A0421	conserved hypothetical protein	417115	417447	Hypothetical proteins
II	BMA_A0422	cupin domain protein	417841	418203	Unknown function
II	BMA_A0423	putative lipoprotein NlpD	418482	419246	Cell envelope
II	BMA_A0424	putative membrane protein	420012	419377	Cell envelope
II	BMA_A0425	nickel-dependent hydrogenases b-type cytochrome subunit	420281	420817	Energy metabolism
II	BMA_A0426	conserved hypothetical protein	420978	421373	Hypothetical proteins
II	BMA_A0427	TonB-dependent copper receptor	421461	423698	Transport and binding proteins
II	BMA_A0428	lipase, authentic frameshift	424022	425115	Fatty acid and phospholipid metabolism
II	BMA_A0429	lipase chaperone	425119	426153	Protein fate
II	BMA_A0430	transcriptional regulator, GntR family/aminotransferase family protein	427775	426255	Regulatory functions
II	BMA_A0431	polyamine ABC transporter, ATP-binding protein	427893	428993	Transport and binding proteins

II	BMA_A0432	putative polyamine ABC transporter, periplasmic polyamine-binding protein	429068	430114	Transport and binding proteins
II	BMA_A0433	putative polyamine ABC transporter, permease protein	430215	431474	Transport and binding proteins
II	BMA_A0434	spermidine/putrescine ABC transporter, permease protein, authentic frameshift	431475	432331	Transport and binding proteins
II	BMA_A0554	di-haem cytochrome c peroxidase family protein, authentic frameshift	563346	564771	Energy metabolism
II	BMA_A0557	arsenical-resistance protein	568753	567683	Cellular processes; Transport and binding proteins
II	BMA_A0558	arsenate reductase	569310	568816	Cellular processes
II	BMA_A0559	glyoxalase family protein	569851	569345	Unknown function
II	BMA_A0560	arsenical resistance transcriptional regulator	570156	569821	Regulatory functions
II	BMA_A0561	hypothetical protein	571485	570628	
II	BMA_A0562	hypothetical protein	572060	571626	
II	BMA_A0563	NADPH-dependent FMN reductase family protein	573706	572435	Unknown function
II	BMA_A0564	glycine betaine/L-proline ABC transporter, ATP-binding protein	573886	575055	Transport and binding proteins
II	BMA_A0565	glycine betaine/L-proline ABC transporter, permease protein	575048	575950	Transport and binding proteins
II	BMA_A0566	transcriptional regulator, AraC family	576073	577071	Regulatory functions
II	BMA_A0567	glycine betaine/L-proline ABC transporter, periplasmic glycine betaine/L-proline-binding protein	577172	578122	Transport and binding proteins
II	BMA_A0568	beta-hydroxylase, aspartyl/asparaginyl family	578505	579404	Energy metabolism
II	BMA_A0568.1	hypothetical protein	579395	579577	
II	BMA_A0570	hypothetical protein	579684	580154	
II	BMA_A0571	beta-hydroxylase, aspartyl/asparaginyl family	580977	580285	Energy metabolism
II	BMA_A0572	NAD-dependent epimerase/dehydratase family protein	582936	581965	Energy metabolism
II	BMA_A0573	hydroxypyruvate isomerase	583740	582964	Energy metabolism
II	BMA_A0574	major facilitator family transporter	585144	583822	Transport and binding proteins
II	BMA_A0575	class II aldolase/adducin domain protein	585889	585248	Unknown function
II	BMA_A0576	conserved hypothetical protein	587253	585886	Hypothetical proteins
II	BMA_A0577	3-hydroxyisobutyrate dehydrogenase family protein	588166	587276	Unknown function
II	BMA_A0578	transcriptional regulator, GntR family	588949	588245	Regulatory functions
II	BMA_A0579	HAD hydrolase, family IA	589140	589754	Unknown function
II	BMA_A0580	cupin domain protein	589849	590331	Unknown function
II	BMA_A0581	hypothetical protein	590288	590827	
II	BMA_A0582	conserved domain protein	591873	590758	Hypothetical proteins
II	BMA_A0586	hypothetical protein	594544	594675	

II	BMA_A0587	putative outer membrane porin	594954	596051	Transport and binding proteins
II	BMA_A0588	hypothetical protein	596609	596346	
II	BMA_A0589	conserved hypothetical protein	597041	596670	Hypothetical proteins
II	BMA_A0590	transcriptional regulator, LuxR family	597981	597208	Regulatory functions
II	BMA_A0591	amino acid permease	598130	599539	Transport and binding proteins
II	BMA_A0592	peptidase, T4 family	599581	600711	Protein fate
II	BMA_A0593	histidine acid phosphatase family protein	602348	600702	Unknown function
II	BMA_A0594	hypothetical protein	602843	602523	
II	BMA_A0595	putative fusaric acid resistance protein	605105	602868	Cellular processes
II	BMA_A0596	transcriptional regulator, LysR family	605316	606326	Regulatory functions
II	BMA_A0597	hypothetical protein	606342	606611	
II	BMA_A0598	hypothetical protein	607347	606829	
II	BMA_A0599	outer membrane porin	607450	608604	Transport and binding proteins
II	BMA_A0600	glyoxalase family protein	608718	609182	Unknown function
II	BMA_A0601	cytosine permease	609732	610994	Transport and binding proteins
II	BMA_A0602	putative purine catabolism transcriptional regulator	610999	612231	Purines, pyrimidines, nucleosides, nucleotides; Regulatory functions
II	BMA_A0603	cytosine deaminase	612231	613487	Purines, pyrimidines, nucleosides, nucleotides
II	BMA_A0604	major facilitator family transporter	614891	613554	Transport and binding proteins
II	BMA_A0605	conserved hypothetical protein	616019	615087	Hypothetical proteins
II	BMA_A0606	oxidoreductase, short-chain dehydrogenase/reductase family	616858	616061	Unknown function
II	BMA_A0607	transcriptional regulator, GntR family	617689	616973	Regulatory functions
II	BMA_A0608	chloride transporter, chloride channel (ClC) family	618036	619382	Transport and binding proteins
II	BMA_A0609	hypothetical protein	620236	619706	
II	BMA_A0610	di-haem cytochrome c peroxidase family protein	621948	620338	Energy metabolism
II	BMA_A0611	phosphoesterase family protein	623619	621952	Unknown function
II	BMA_A0612	putative acetylesterase	625168	624188	Unknown function
II	BMA_A0613	conserved hypothetical protein	625227	625550	Hypothetical proteins
II	BMA_A0614	conserved domain protein	625707	625540	Hypothetical proteins
II	BMA_A0615	DNA-binding response regulator, LuxR family	626342	625704	Regulatory functions; Signal transduction
II	BMA_A0616	hypothetical protein	626575	626339	
II	BMA_A0617	hypothetical protein	627250	626636	
II	BMA_A0618	hypothetical protein	627370	627738	
II	BMA_A0619	transcriptional regulator, MarR family	628340	628822	Regulatory functions
II	BMA_A0621	putative arabinose ABC transporter, permease protein, authentic frameshift	629562	630396	Transport and binding proteins
II	BMA_A0620	LysR substrate-binding domain family protein, degenerate	629707	629095	Unknown function

II	BMA_A0622	senescence marker protein-30 family protein	630663	631598	Unknown function
II	BMA_A0623	hypothetical protein	631730	632140	
II	BMA_A0624	conserved hypothetical protein	632286	632702	Hypothetical proteins
II	BMA_A0625	conserved hypothetical protein	633228	632788	Hypothetical proteins
II	BMA_A0626	hypothetical protein	633932	633321	
II	BMA_A0628	hypothetical protein	634641	634919	
II	BMA_A0629	p-hydroxycinnamoyl CoA hydratase/lyase	635023	635850	Energy metabolism
II	BMA_A0630	vanillin dehydrogenase	636017	637465	Energy metabolism
II	BMA_A0631	putative feruloyl-CoA synthetase, authentic frameshift	637576	639307	Energy metabolism
II	BMA_A0632	MFS transporter, aromatic acid:H+ symporter (AAHS) family	639559	640764	Transport and binding proteins
II	BMA_A0633	outer membrane porin	641298	642437	Transport and binding proteins
II	BMA_A0634	tannase/feruloyl esterase family protein	642528	644234	Energy metabolism
II	BMA_A0635	hypothetical protein	644247	644459	
II	BMA_A0636	transcriptional regulator, IclR family	645323	644499	Regulatory functions
II	BMA_A0637	ABC transporter, carbohydrate uptake transporter-2 (CUT2) family, periplasmic sugar-binding protein	645512	646525	Transport and binding proteins
II	BMA_A0638	ABC transporter, carbohydrate uptake transporter-2 (CUT2) family, ATP-binding protein	646522	648078	Transport and binding proteins
II	BMA_A0639	ABC transporter, carbohydrate uptake transporter-2 (CUT2) family, permease protein	648068	649093	Transport and binding proteins
II	BMA_A0640	putative D-galactarate dehydratase/altronate dehydratase, authentic frameshift	649090	650767	Energy metabolism
II	BMA_A0641	amidohydrolase family protein	651039	651878	Unknown function
II	BMA_A0642	oxidoreductase, short chain dehydrogenase/reductase family	652018	652761	Unknown function
II	BMA_A0643	fumarylacetoacetate hydrolase family protein	652837	653679	Unknown function
II	BMA_A0645	conserved hypothetical protein	654643	654981	Hypothetical proteins
II	BMA_A0646	hypothetical protein	655441	655091	
II	BMA_A0650	hypothetical protein	663183	663806	
II	BMA_A0651	H-NS histone family protein	664187	663846	DNA metabolism; Regulatory functions
II	BMA_A0652	transcriptional regulator, IclR family	665118	664378	Regulatory functions
II	BMA_A0653	hypothetical protein	665652	665233	
II	BMA_A0654	cyclic diguanylate phosphodiesterase	666756	665713	Regulatory functions; Signal transduction
II	BMA_A0655	hypothetical protein	666866	666970	
II	BMA_A0656	hypothetical protein	667015	666899	
II	BMA_A0657	hypothetical protein	667481	667311	
II	BMA_A0658	transcriptional regulator, TetR family	668424	667810	Regulatory functions
II	BMA_A0659	conserved hypothetical protein	668539	668976	Hypothetical proteins

II	BMA_A0662	hemolysin III	670744	671364	Cellular processes
II	BMA_A0661	hypothetical protein	670766	670359	
II	BMA_A0663	hypothetical protein	672438	671422	
II	BMA_A0664	putative diguanylate cyclase/putative cyclic diguanylate phosphodiesterase	674501	672435	Unknown function
II	BMA_A0665	putative leucine-responsive regulatory protein	675295	674828	Regulatory functions
II	BMA_A0666	aromatic amino acid transport protein AroP	675628	677013	Transport and binding proteins
II	BMA_A0667	aromatic-amino-acid aminotransferase	677045	678247	Amino acid biosynthesis
II	BMA_A0668	conserved hypothetical protein	678579	678968	Hypothetical proteins
II	BMA_A0669	hypothetical protein	678965	679756	
II	BMA_A0670	hypothetical protein	679774	680052	
II	BMA_A0671	acetyltransferase, GNAT family	680112	680726	Unknown function
II	BMA_A0672	oxidoreductase, 2OG-Fe(II) oxygenase family	681541	680891	Unknown function
II	BMA_A0673	conserved hypothetical protein	682798	681653	Hypothetical proteins
II	BMA_A0673.1	hypothetical protein	683297	683058	
II	BMA_A0673.2	hypothetical protein	683389	683934	
II	BMA_A0676	hypothetical protein	683973	684350	
II	BMA_A0677	sensor histidine kinase	684742	686076	Regulatory functions; Signal transduction
II	BMA_A0678	response regulator	686087	686455	Regulatory functions; Signal transduction
II	BMA_A0679	phosphatase, CheC family	686439	687059	Regulatory functions; Signal transduction
II	BMA_A0681	alpha-2-macroglobulin family protein	696097	689930	Unknown function
II	BMA_A0682	hypothetical protein	696459	696094	
II	BMA_A0683	conserved hypothetical protein	696578	697321	Hypothetical proteins
II	BMA_A0684	conserved hypothetical protein	697556	698131	Hypothetical proteins
II	BMA_A0685	conserved hypothetical protein	698271	698741	Hypothetical proteins
II	BMA_A0686	leucyl-tRNA synthetase, authentic frameshift	698738	700940	Protein synthesis
II	BMA_A0687	conserved hypothetical protein	700937	701857	Hypothetical proteins
II	BMA_A0688	conserved hypothetical protein	701860	702762	Hypothetical proteins
II	BMA_A0689	conserved hypothetical protein	702759	704372	Hypothetical proteins
II	BMA_A0690	dioxygenase, TauD/TfdA family	704365	705258	Unknown function
II	BMA_A0691	conserved hypothetical protein	705929	705222	Hypothetical proteins
II	BMA_A0692	putative isopenicillin N epimerase	705941	707098	Cellular processes
II	BMA_A0693	hypothetical protein	707868	707347	
II	BMA_A0694	putative microbial collagenase	708147	710045	Protein fate
II	BMA_A0695	hypothetical protein	710139	710744	
II	BMA_A0696	FAD-dependent oxidoreductase	712484	711318	Unknown function
II	BMA_A0697	hypothetical protein	713003	712611	
II	BMA_A0698	DNA-binding protein	713810	713082	Unknown function
II	BMA_A0699	hypothetical protein	714197	714385	
II	BMA_A0699.1	hypothetical protein	715206	714643	
II	BMA_A0701	conserved hypothetical protein	715321	715611	Hypothetical proteins
II	BMA_A0702	conserved hypothetical protein	715673	715909	Hypothetical proteins

II	BMA_A0703	DNA-binding response regulator, LuxR family	716635	715988	Regulatory functions; Signal transduction
II	BMA_A0704	hypothetical protein	716835	716996	
II	BMA_A0705	universal stress protein family	717197	717655	Cellular processes
II	BMA_A0706	universal stress family protein	718858	718022	Cellular processes
II	BMA_A0707	universal stress protein family	719352	718858	Cellular processes
II	BMA_A0708	universal stress family protein	720373	719534	Cellular processes
II	BMA_A0709	alcohol dehydrogenase, zinc-containing	720623	721663	Energy metabolism
II	BMA_A0710	hypothetical protein	721974	721828	
II	BMA_A0711	dedA family protein	722144	723190	Unknown function
II	BMA_A0712	major facilitator family transporter	723379	724587	Transport and binding proteins
II	BMA_A0713	hypothetical protein	724855	724670	
II	BMA_A0714	oxidoreductase, short-chain dehydrogenase/reductase family	724992	725768	Unknown function
II	BMA_A0715	conserved hypothetical protein	725784	726653	Hypothetical proteins
II	BMA_A0716	hypothetical protein	727526	727059	
II	BMA_A0717	amino acid permease	727563	728906	Transport and binding proteins
II	BMA_A0718	hypothetical protein	729010	729522	
II	BMA_A0719	DNA-binding protein	729744	729502	Unknown function
II	BMA_A0720	H-NS histone family protein	729889	730185	DNA metabolism; Regulatory functions
II	BMA_A0721	acyl-CoA dehydrogenase domain protein	731895	730579	Fatty acid and phospholipid metabolism
II	BMA_A0722	putative membrane protein	732895	732317	Cell envelope
II	BMA_A0723	conserved hypothetical protein	733816	732932	Hypothetical proteins
II	BMA_A0814	glutaminase A	841443	840529	Energy metabolism
II	BMA_A0815	DNA-binding response regulator	841982	842692	Regulatory functions; Signal transduction
II	BMA_A0816	sensor histidine kinase	843080	844195	Regulatory functions; Signal transduction
II	BMA_A0818	phospholipase, patatin family	846718	844532	Fatty acid and phospholipid metabolism
II	BMA_A0819	putative rod shape-determining protein MreB	847630	848694	Cell envelope
II	BMA_A0820	cytochrome c4 family protein	849075	848746	Energy metabolism
II	BMA_A0821	HD domain protein	849077	849781	Unknown function
II	BMA_A0822	amylo-alpha-1,6-glucosidase	850074	852203	Energy metabolism
II	BMA_A0823	hypothetical protein	852247	852822	
II	BMA_A0825	hypothetical protein	853843	854121	
II	BMA_A0824	conserved hypothetical protein, authentic frameshift	853870	852798	Hypothetical proteins
II	BMA_A0826	glyoxalase family protein	854150	854626	Unknown function
II	BMA_A0827	glutathione S-transferase family protein	854925	855584	Central intermediary metabolism
II	BMA_A0828	conserved hypothetical protein	855625	856335	Hypothetical proteins
II	BMA_A0829	sensor histidine kinase	859594	858215	Regulatory functions; Signal transduction

II	BMA_A0830	DNA-binding response regulator	860253	859591	Regulatory functions; Signal transduction
II	BMA_A0831	phosphosugar-binding transcriptional regulator, RpiR family	860414	861274	Regulatory functions
II	BMA_A0832	hypothetical protein	862085	861567	
II	BMA_A0833	putative immunity protein	862484	862146	Mobile and extrachromosomal elements
II	BMA_A0834	rhodanese-like domain protein	863041	862574	Unknown function
II	BMA_A0835	transcriptional regulator, AraC family	863148	864104	Regulatory functions
II	BMA_A0836	peptidase, M20/M25/M40 family	865220	866671	Protein fate
II	BMA_A0837	TfoX domain protein	866781	867149	Unknown function
II	BMA_A0838	hypothetical protein	867194	867688	
II	BMA_A0839	NnrU protein	867670	868254	Cell envelope
II	BMA_A0840	conserved hypothetical protein	868281	868538	Hypothetical proteins
II	BMA_A0841	conserved hypothetical protein, degenerate	868531	868945	Hypothetical proteins
II	BMA_A0842	hypothetical protein	868946	869047	
II	BMA_A0843	hypothetical protein	869058	869363	
II	BMA_A0844	transposase, degenerate	869333	869581	Mobile and extrachromosomal elements
II	BMA_A0873	conserved hypothetical protein	895207	895533	Hypothetical proteins
II	BMA_A0874	conserved hypothetical protein	896391	895546	Hypothetical proteins
II	BMA_A0875	oxidoreductase, aldo/keto reductase family	897511	896540	Unknown function
II	BMA_A0877	hypothetical protein	898513	898647	
II	BMA_A1001	hypothetical protein	1029802	1030824	
II	BMA_A1002	putative toxin secretion ABC transporter, membrane fusion protein	1030837	1032132	Cellular processes; Transport and binding proteins
II	BMA_A1003	ompW family protein	1032805	1033638	Cell envelope
II	BMA_A1007	transcriptional regulator, TetR family	1037027	1036311	Regulatory functions
II	BMA_A1008	amine oxidase, flavin-containing	1037106	1038404	Energy metabolism
II	BMA_A1011	hypothetical protein	1040916	1041056	
II	BMA_A1010	conserved hypothetical protein	1040917	1040327	Hypothetical proteins
II	BMA_A1012	hypothetical protein	1041031	1041357	
II	BMA_A1013	RNA polymerase sigma-70 factor, ECF subfamily	1042201	1041695	Transcription
II	BMA_A1014	conserved hypothetical protein	1042497	1042249	Hypothetical proteins
II	BMA_A1015	hypothetical protein	1042719	1042603	
II	BMA_A1016	AMP-binding domain protein	1042848	1044647	Unknown function
II	BMA_A1017	putative acyl-CoA dehydrogenase	1044644	1046404	Unknown function
II	BMA_A1018	acyl-CoA dehydrogenase, C-terminal domain	1046401	1048194	Unknown function
II	BMA_A1019	phosphopantetheine attachment site domain protein	1048205	1048495	Unknown function
II	BMA_A1020	conserved hypothetical protein	1048930	1049733	Hypothetical proteins
II	BMA_A1022	putative polyketide synthase	1059105	1061756	Cellular processes
II	BMA_A1122	putative outer membrane porin	1175037	1173835	Transport and binding proteins
II	BMA_A1124	hypothetical protein	1176775	1177833	

II	BMA_A1125	MOSC domain protein	1178265	1179134	Unknown function
II	BMA_A1126	sensor histidine kinase	1181268	1179763	Regulatory functions; Signal transduction
II	BMA_A1127	DNA-binding response regulator TctD	1181936	1181265	Regulatory functions; Signal transduction
II	BMA_A1128	ABC transporter, periplasmic substrate-binding protein	1182065	1183153	Transport and binding proteins
II	BMA_A1129	ABC transporter, permease protein, authentic frameshift	1183254	1185021	Transport and binding proteins
II	BMA_A1131	putative outer membrane porin	1186559	1187650	Transport and binding proteins
II	BMA_A1132	oxidoreductase, zinc-binding dehydrogenase family	1188708	1187743	Unknown function
II	BMA_A1133	transcriptional regulator, AraC family	1188985	1189827	Regulatory functions
II	BMA_A1134	2-dehydro-3-deoxyglucarate aldolase, authentic frameshift	1190569	1189776	Energy metabolism
II	BMA_A1135	2-oxo-hepta-3-ene-1,7-dioic acid hydratase	1191382	1190579	Energy metabolism
II	BMA_A1136	5-carboxymethyl-2-hydroxymuconate delta isomerase	1191797	1191405	Energy metabolism
II	BMA_A1137	3,4-dihydroxyphenylacetate 2,3-dioxygenase	1192703	1191855	Energy metabolism
II	BMA_A1138	5-carboxymethyl-2-hydroxymuconate semialdehyde dehydrogenase	1194236	1192773	Energy metabolism
II	BMA_A1139	4-hydroxyphenylacetate degradation bifunctional isomerase/decarboxylase, C-terminal subunit	1195024	1194260	Energy metabolism
II	BMA_A1140	4-hydroxyphenylacetate degradation bifunctional isomerase/decarboxylase, N-terminal subunit	1195668	1195021	Energy metabolism
II	BMA_A1141	homoprotocatechuate degradation operon regulator, HpaR	1195876	1196313	Central intermediary metabolism; Regulatory functions
II	BMA_A1142	NAD-dependent epimerase/dehydratase family protein	1197553	1196357	Energy metabolism
II	BMA_A1143	mipA family protein	1198314	1197568	Unknown function
II	BMA_A1144	DNA-binding response regulator	1198555	1199277	Regulatory functions; Signal transduction
II	BMA_A1145	sensor histidine kinase	1199278	1200606	Regulatory functions; Signal transduction
II	BMA_A1146	conserved hypothetical protein	1202285	1200882	Hypothetical proteins
II	BMA_A1147	acyl-CoA dehydrogenase domain protein	1203575	1202373	Unknown function
II	BMA_A1149	hypothetical protein	1204829	1205029	
II	BMA_A1148	acyl-CoA dehydrogenase domain protein	1204830	1203592	Fatty acid and phospholipid metabolism
II	BMA_A1150	hypothetical protein	1205235	1205591	
II	BMA_A1151	conserved hypothetical protein	1206415	1206023	Hypothetical proteins
II	BMA_A1152	hypothetical protein	1206475	1206651	
II	BMA_A1153	transcriptional regulator, AraC family	1207619	1206642	Regulatory functions
II	BMA_A1154	aldehyde dehydrogenase subunit III	1207826	1208287	Energy metabolism

II	BMA_A1154.1	isoquinoline 1-oxidoreductase, beta subunit	1208290	1210521	Central intermediary metabolism
II	BMA_A1157	transcriptional regulatory protein, C terminal domain, truncation	1211395	1211610	Regulatory functions
II	BMA_A1156	hypothetical protein	1211396	1211058	
II	BMA_A1159	autoinducer-binding transcriptional regulator, LuxR family	1214674	1213850	Regulatory functions; Signal transduction
II	BMA_A1160	hypothetical protein	1214925	1215065	
II	BMA_A1161	transcriptional regulator, LuxR family	1215198	1216088	Regulatory functions
II	BMA_A1162	hypothetical protein	1216540	1216142	
II	BMA_A1163	cytochrome c oxidase, subunit III family protein	1217133	1216504	Energy metabolism
II	BMA_A1164	putative membrane protein	1217854	1217162	Cell envelope
II	BMA_A1165	hypothetical protein	1217899	1218297	
II	BMA_A1166	trehalase	1220069	1218375	Energy metabolism
II	BMA_A1168	transporter, major facilitator family	1220747	1222144	Transport and binding proteins
II	BMA_A1167	hypothetical protein	1220748	1220191	
II	BMA_A1169	conserved hypothetical protein	1222561	1223790	Hypothetical proteins
II	BMA_A1170	conserved hypothetical protein	1224200	1223982	Hypothetical proteins
II	BMA_A1172	hypothetical protein	1224684	1224899	
II	BMA_A1171	conserved hypothetical protein, authentic frameshift	1224708	1224197	Hypothetical proteins
II	BMA_A1413	ISBma3, transposase, truncation	1518817	1519283	Mobile and extrachromosomal elements
II	BMA_A1414	conserved hypothetical protein	1519497	1519766	Hypothetical proteins
II	BMA_A1415	transcriptional regulator, AraC family	1520836	1519964	Regulatory functions
II	BMA_A1416	pyridine nucleotide-disulphide oxidoreductase	1522164	1520872	Unknown function
II	BMA_A1417	2Fe-2S iron-sulfur cluster-binding protein	1522394	1522161	Energy metabolism
II	BMA_A1418	oxidoreductase, FAD-binding family protein	1523533	1522391	Unknown function
II	BMA_A1419	putative proline racemase	1524462	1523530	Energy metabolism
II	BMA_A1420	dihydrodipicolinate synthetase family protein	1524713	1525633	Unknown function
II	BMA_A1421	amino acid permease	1526094	1527653	Transport and binding proteins
II	BMA_A1422	aldehyde dehydrogenase (NAD) family protein	1527729	1529321	Energy metabolism
II	BMA_A1423	malate/L-lactate dehydrogenase family protein	1529314	1530348	Unknown function
II	BMA_A1424	major facilitator family transporter	1530690	1531982	Transport and binding proteins
II	BMA_A1425	putative methylenomycin A resistance protein	1532250	1533785	Cellular processes; Transport and binding proteins
II	BMA_A1426	DoxD-like family protein	1534418	1533981	Unknown function
II	BMA_A1427	hypothetical protein	1534774	1534415	

II	BMA_A1428	transporter, lactate permease (LctP) family	1536465	1534747	Transport and binding proteins
II	BMA_A1429	conserved hypothetical protein	1537558	1536833	Hypothetical proteins
II	BMA_A1430	iron-sulfur cluster-binding protein	1539003	1537555	Energy metabolism
II	BMA_A1431	cysteine-rich domain protein	1539806	1539000	Unknown function
II	BMA_A1432	transcriptional regulator, GntR family	1540208	1540927	Regulatory functions
II	BMA_A1433	hypothetical protein	1540981	1541547	
II	BMA_A1435	hypothetical protein	1541912	1542670	
II	BMA_A1434	hypothetical protein	1541913	1541560	
II	BMA_A1436	acyl-CoA dehydrogenase C-terminal domain protein	1543953	1542772	Unknown function
II	BMA_A1436.1	hypothetical protein	1544674	1544153	
II	BMA_A1438	ABC transporter, methionine uptake transporter (MUT) family, permease protein	1545365	1544709	Transport and binding proteins
II	BMA_A1439	ABC transporter, methionine uptake transporter (MUT) family, ATP-binding protein	1546572	1545358	Transport and binding proteins
II	BMA_A1440	conserved hypothetical protein	1547987	1546569	Hypothetical proteins
II	BMA_A1441	ABC transporter, methionine uptake transporter (MUT) family, periplasmic amino-acid binding protein	1548820	1547981	Transport and binding proteins
II	BMA_A1442	hypothetical protein	1549094	1548834	
II	BMA_A1443	autoinducer-binding transcriptional regulator BmaR4	1549866	1549150	Cellular processes; Regulatory functions; Signal transduction
II	BMA_A1444	hypothetical protein	1550246	1550683	
II	BMA_A1445	hypothetical protein	1550769	1550990	
II	BMA_A1446	thiotemplate mechanism natural product synthetase	1551057	1559576	Cellular processes
II	BMA_A1447	conserved domain protein	1559676	1560194	Hypothetical proteins
II	BMA_A1448	hypothetical protein	1560398	1560982	
II	BMA_A1449	putative peptide synthase regulatory protein	1561220	1562245	Regulatory functions
II	BMA_A1450	putative adenosylmethionine-8-amino-7-oxononanoate aminotransferase	1562254	1563675	Biosynthesis of cofactors, prosthetic groups, carriers
II	BMA_A1451	aldehyde dehydrogenase family protein	1563669	1565081	Energy metabolism
II	BMA_A1451.1	polyketide synthase	1565069	1577383	Cellular processes
II	BMA_A1453	putative ketol-acid reductoisomerase	1577389	1578288	Amino acid biosynthesis
II	BMA_A1454	FkbH domain protein	1578285	1579766	Unknown function
II	BMA_A1456	AMP-binding domain protein	1581052	1582911	Unknown function
II	BMA_A1457	putative lipoprotein	1582979	1583410	Cell envelope
II	BMA_A1458	malonyl CoA-acyl carrier protein transacylase	1583411	1584310	Fatty acid and phospholipid metabolism
II	BMA_A1459	AMP-binding domain protein	1584327	1586192	Unknown function
II	BMA_A1460	transporter, auxin efflux carrier (AEC) family	1586645	1587544	Transport and binding proteins
II	BMA_A1461	acetyltransferase, GNAT family	1588202	1587732	Unknown function
II	BMA_A1462	transcriptional regulator, GntR family	1588358	1589017	Regulatory functions

II	BMA_A1463	transporter, dicarboxylate/amino acid:cation (Na+ or H+) symporter (DAACS) family	1589001	1590242	Transport and binding proteins
II	BMA_A1464	outer membrane efflux protein OprC	1592660	1591128	Cellular processes; Transport and binding proteins
II	BMA_A1465	multidrug efflux pump BpeF	1595856	1592671	Cellular processes; Transport and binding proteins
II	BMA_A1466	multidrug efflux pump BpeE	1597147	1595918	Cellular processes; Transport and binding proteins
II	BMA_A1467	putative esterase	1598147	1597197	Fatty acid and phospholipid metabolism
II	BMA_A1468	transcriptional regulator, LysR family	1598240	1599244	Regulatory functions
II	BMA_A1469	hypothetical protein	1599407	1599249	
II	BMA_A1470	hypothetical protein	1599529	1600536	
II	BMA_A1471	purine nucleoside permease family protein	1601763	1600702	Transport and binding proteins
II	BMA_A1472	hypothetical protein	1602354	1601776	
II	BMA_A1473	selenide, water dikinase	1604186	1603122	Protein synthesis
II	BMA_A1474	putative membrane protein	1604404	1605237	Cell envelope
II	BMA_A1475	porin, degenerate	1605810	1605371	Transport and binding proteins
II	BMA_A1476	putative N-formylglutamate amidohydrolase	1606153	1607061	Energy metabolism
II	BMA_A1477	transcriptional regulator, IclR family	1607058	1607825	Regulatory functions
II	BMA_A1478	C4-dicarboxylate anaerobic carrier family protein	1607909	1609321	Transport and binding proteins
II	BMA_A1478.1	transcriptional regulator, GntR family/aminotransferase	1610978	1609338	Regulatory functions
II	BMA_A1480	4-aminobutyrate transaminase	1611120	1612403	Central intermediary metabolism
II	BMA_A1482	hypothetical protein	1614705	1613905	
II	BMA_A1483	hypothetical protein	1615059	1615640	
II	BMA_A1484	conserved hypothetical protein, authentic frameshift	1615637	1617666	Hypothetical proteins
II	BMA_A1486	O-methyltransferase	1618084	1619100	Unknown function
II	BMA_A1485	hypothetical protein	1618085	1617867	
II	BMA_A1488	hypothetical protein	1620641	1621096	
II	BMA_A1489	hypothetical protein	1621232	1621414	
II	BMA_A1491	beta-ketoadipate pathway transcriptional regulator, PcaR/PcaU/PobR family	1622956	1623741	Regulatory functions
II	BMA_A1492	transcriptional regulator, TetR family	1624102	1624821	Regulatory functions
II	BMA_A1493	major facilitator family transporter	1626686	1625370	Transport and binding proteins
II	BMA_A1494	hippurate hydrolase	1628147	1626960	Central intermediary metabolism
II	BMA_A1495	transcriptional regulator, LysR family	1628283	1629182	Regulatory functions

II	BMA_A1496	putative lysine-arginine-ornithine-binding periplasmic protein	1629416	1630198	Transport and binding proteins
II	BMA_A1497	hypothetical protein	1630233	1630517	
II	BMA_A1498	putative O-antigen acetylase	1632613	1630514	Cell envelope
II	BMA_A1499	succinylglutamate desuccinylase / aspartoacylase family protein	1632735	1634015	Unknown function
II	BMA_A1500	hypothetical protein	1634258	1634136	
II	BMA_A1501	D-methionine-binding lipoprotein metQ	1635297	1634488	Cell envelope; Transport and binding proteins
II	BMA_A1502	porin, interruption-N	1635475	1636311	Transport and binding proteins
II	BMA_A1505	porin, interruption-C	1637588	1637989	Transport and binding proteins
II	BMA_A1507	hypothetical protein	1639902	1639615	
II	BMA_A1508	transcriptional regulator, LysR family	1640911	1639940	Regulatory functions
II	BMA_A1509	pirin domain protein	1641034	1641906	Unknown function
II	BMA_A1510	conserved hypothetical protein	1642025	1642510	Hypothetical proteins
II	BMA_A1511	conserved hypothetical protein	1642771	1642917	Hypothetical proteins
II	BMA_A1514	hypothetical protein	1644440	1644742	
II	BMA_A1515	hypothetical protein	1645619	1645176	
II	BMA_A1516	ISBma5, transposase, authentic frameshift	1645780	1646659	Mobile and extrachromosomal elements
II	BMA_A1517	transcriptional regulator, AraC family	1647593	1646532	Cellular processes; Regulatory functions
II	BMA_A1518	conserved hypothetical protein	1648419	1647976	Hypothetical proteins
II	BMA_A1518.1	DNA-binding response regulator	1649346	1648675	Regulatory functions; Signal transduction
II	**BMA_A1520**	**type III secretion chaperone BicP**	1650045	1649590	Cellular processes; Protein fate
II	BMA_A1522	hypothetical protein	1651728	1652267	
II	BMA_A1521	virulence protein IcsB	1651735	1650197	Unknown function
II	BMA_A1523	putative guanine nucleotide exchange factor sopE	1652361	1653146	Cellular processes; Transport and binding proteins; Mobile and extrachromosomal elements
II	BMA_A1524	BapC protein	1654160	1653597	Cell envelope
II	BMA_A1525	acyl carrier protein BapB	1654438	1654157	Fatty acid and phospholipid metabolism
II	BMA_A1526	BapA protein	1655134	1654622	Unknown function
II	BMA_A1528	BprD protein	1658087	1657230	Cellular processes; Protein fate
II	BMA_A1529	H-NS histone family protein	1658514	1658224	DNA metabolism; Regulatory functions
II	BMA_A1530	type III secretion target BipC	1659907	1658648	Cellular processes
II	BMA_A1531	BipB protein	1661811	1659949	Cellular processes
II	**BMA_A1532**	**type III secretion chaperone BicA**	1662375	1661830	Cellular processes; Protein fate
II	**BMA_A1533**	**type III secretion system protein BsaZ**	1663706	1662471	Cellular processes; Protein fate

II	**BMA_A1534**	**type III secretion system protein BsaY**	1664480	1663710	Cellular processes; Protein fate
II	**BMA_A1535**	**type III secretion system protein BsaX**	1664753	1664499	Cellular processes; Protein fate
II	**BMA_A1536**	**type III secretion system protein BsaW**	1665469	1664789	Cellular processes; Protein fate
II	**BMA_A1537**	**type III secretion system protein BsaV**	1666442	1665459	Cellular processes; Protein fate
II	BMA_A1538	BsaU protein	1667686	1666439	Unknown function
II	BMA_A1539	surface presentation of antigens	1668128	1667664	Unknown function
II	**BMA_A1540**	**type III secretion system protein BsaS**	1669435	1668125	Cellular processes; Protein fate
II	**BMA_A1541**	**type III secretion system protein BsaR**	1669839	1669432	Cellular processes; Protein fate
II	**BMA_A1542**	**type III secretion system protein BsaQ**	1671923	1669851	Cellular processes; Protein fate
II	**BMA_A1543**	**type III secretion system protein BsaP**	1673080	1671959	Cellular processes; Protein fate
II	**BMA_A1544**	**type III secretion system protein BsaO**	1674897	1673077	Cellular processes; Protein fate
II	**BMA_A1545**	**type III secretion system transcriptional regulator BsaN**	1675726	1674968	Cellular processes; Regulatory functions
II	BMA_A1546	hypothetical protein	1675914	1676123	
II	**BMA_A1547**	**type III secretion system protein BsaM**	1676120	1677406	Cellular processes; Protein fate
II	**BMA_A1548**	**type III secretion system protein BsaL**	1677403	1677672	Cellular processes; Protein fate
II	BMA_A1549	putative type III secretion system protein	1677727	1678029	Cellular processes; Protein fate
II	**BMA_A1550**	**type III secretion system BasJ**	1678034	1678984	Cellular processes; Protein fate
II	**BMA_A1551**	**type III secretion apparatus protein OrgA/MxiK**	1678981	1679568	Cellular processes; Protein fate
II	**BMA_A1552**	**type III secretion apparatus protein, HrpE/YscL family**	1679537	1680331	Cellular processes; Protein fate
II	BMA_A1553	hypothetical protein	1680706	1680272	
II	BMA_A1554	hypothetical protein	1680855	1681046	
II	BMA_A1555	hypothetical protein	1681389	1681261	
II	BMA_A1556	putative transcriptional regulator	1681430	1682296	Regulatory functions
II	BMA_A1557	hypothetical protein	1682263	1682748	
II	BMA_A1558	hypothetical protein	1682897	1683487	
II	BMA_A1559	thermolysin metallopeptidase	1683758	1685455	Protein fate
II	BMA_A1560	hypothetical protein	1686049	1685930	
II	BMA_A1561	transcriptional regulator, MarR family	1687199	1686561	Regulatory functions
II	BMA_A1562	glyoxalase family protein	1687376	1687834	Unknown function
II	BMA_A1564	transcriptional regulator, LysR family	1690965	1690078	Regulatory functions
II	BMA_A1565	FMN-dependent dehydrogenase	1691066	1692289	Unknown function
II	BMA_A1566	serine carboxypeptidase family protein	1694077	1692413	Unknown function
II	BMA_A1567	hypothetical protein	1694751	1694245	

II	BMA_A1568	serine protease, subtilase family	1696408	1694834	Protein fate
II	BMA_A1569	translocator protein, LysE family	1697453	1696839	Transport and binding proteins
II	BMA_A1570	transcriptional regulator, AsnC family	1697527	1698051	Regulatory functions
II	BMA_A1571	conserved hypothetical protein	1698048	1698779	Hypothetical proteins
II	BMA_A1572	phosphate transporter family protein	1699173	1700759	Transport and binding proteins
II	BMA_A1573	conserved hypothetical protein	1700909	1701691	Hypothetical proteins
II	BMA_A1574	AMP-binding domain protein	1702271	1703878	Unknown function
II	BMA_A1575	hypothetical protein	1704472	1704203	
II	BMA_A1576	autoinducer-binding transcriptional regulator BmaR3	1704646	1705338	Cellular processes; Regulatory functions; Signal transduction
II	BMA_A1577	autoinducer synthetase BmaI3	1705664	1706272	Cellular processes; Signal transduction
II	BMA_A1578	oxidoreductase, FAD/FMN-binding	1706605	1707930	Unknown function
II	BMA_A1579	hypothetical protein	1708260	1708069	
II	BMA_A1580	taurine ABC transporter, permease protein	1709133	1708276	Transport and binding proteins
II	BMA_A1581	taurine ABC transporter, ATP-binding protein	1709912	1709130	Transport and binding proteins
II	BMA_A1583	taurine dioxygenase-related protein	1710624	1710935	Unknown function
II	BMA_A1582	taurine ABC transporter, periplasmic taurine-binding protein	1710667	1709933	Transport and binding proteins
II	BMA_A1584	conserved hypothetical protein	1712924	1711368	Hypothetical proteins
II	BMA_A1585	cellulose synthase, catalytic subunit, UDP-forming	1715504	1712958	Cell envelope
II	BMA_A1586	cellulose synthase operon protein YhjQ	1716364	1715501	Cell envelope
II	BMA_A1587	conserved hypothetical protein	1718834	1716570	Hypothetical proteins
II	BMA_A1588	putative cellulose synthase operon protein C	1723239	1718845	Energy metabolism
II	BMA_A1589	cellulase	1724753	1723566	Energy metabolism
II	BMA_A1590	putative cellulose synthase, cyclic di-GMP-binding regulatory subunit	1727475	1725115	Energy metabolism
II	BMA_A1591	hypothetical protein	1727735	1728421	
II	BMA_A1592	putative transcriptionl regulator HipB	1728577	1728981	Regulatory functions
II	BMA_A1593	putative regulatory protein HipA	1728978	1730330	Regulatory functions
II	BMA_A1594	hypothetical protein	1730327	1730899	
II	BMA_A1595	hypothetical protein	1731109	1730900	
II	BMA_A1596	transcriptional regulator, LysR family	1732263	1731349	Regulatory functions
II	BMA_A1597	agmatinase	1732430	1733383	Central intermediary metabolism
II	BMA_A1597.1	hypothetical protein	1733469	1735394	
II	BMA_A1599	hypothetical protein	1735944	1736489	
II	BMA_A1600	conserved hypothetical protein	1736740	1737540	Hypothetical proteins
II	BMA_A1601	hypothetical protein	1739057	1737699	
II	**BMA_A1602**	**type III secretion outer membrane pore, YscC/HrcC family**	1740918	1739119	Cellular processes; Protein fate

II	BMA_A1603	type IV prepilin	1742715	1740988	Cell envelope; Cellular processes
II	BMA_A1604	conserved hypothetical protein	1743194	1742715	Hypothetical proteins
II	BMA_A1605	putative type IV pilus protein	1743788	1743234	Cell envelope
II	BMA_A1606	type IV pilus biogenesis protein	1744897	1743821	Cell envelope; Cellular processes
II	BMA_A1607	type IV pilus protein PilQ	1746497	1744887	Cell envelope; Cellular processes
II	BMA_A1608	putative type IV pilus biogenesis protein PilP	1747018	1746494	Cell envelope
II	BMA_A1609	putative PilO	1748306	1747008	Cell envelope; Cellular processes
II	BMA_A1610	type IVB pilus formation outer membrane protein, R64 PilN family	1750090	1748318	Cell envelope; Cellular processes
II	BMA_A1611	pilL domain protein	1750659	1750090	Unknown function
II	BMA_A1612	putative twitching motility protein PilT	1751051	1752082	Cellular processes
II	**BMA_A1613**	**type II/III secretion system family protein**	1754093	1752303	Protein fate
II	BMA_A1614	DNA-binding response regulator	1754824	1754090	Regulatory functions; Signal transduction
II	BMA_A1615	sensor histidine kinase	1756086	1754890	Regulatory functions; Signal transduction
II	BMA_A1616	hypothetical protein	1756882	1756103	
II	BMA_A1617	putative type III secretion protein HrpB1/HrpK	1756956	1757444	Cellular processes; Protein fate
II	BMA_A1618	conserved hypothetical protein	1758576	1757968	Hypothetical proteins
II	BMA_A1619	hypothetical protein	1759113	1759487	
II	BMA_A1620	conserved hypothetical protein	1759978	1759703	Hypothetical proteins
II	BMA_A1621	regulatory protein HrpB	1761465	1760032	Cellular processes; Regulatory functions
II	BMA_A1622	conserved hypothetical protein	1762132	1761677	Hypothetical proteins
II	BMA_A1623	hypothetical protein	1762413	1762225	
II	BMA_A1624	conserved hypothetical protein	1762828	1762577	Hypothetical proteins
II	**BMA_A1625**	**type III secretion inner membrane protein, authentic frameshift**	1763834	1762891	Cellular processes; Protein fate
II	BMA_A1626	conserved hypothetical protein	1764731	1764030	Hypothetical proteins
II	**BMA_A1627**	**type III secretion inner membrane protein SctS**	1765159	1764896	Cellular processes; Protein fate
II	**BMA_A1628**	**type III secretion inner membrane protein SctR**	1765850	1765200	Cellular processes; Protein fate
II	**BMA_A1629**	**type III secretion inner membrane protein SctQ**	1767135	1765837	Cellular processes; Protein fate
II	**BMA_A1630**	**type III secretion inner membrane protein SctV**	1769843	1767771	Cellular processes; Protein fate
II	**BMA_A1631**	**type III secretion protein, YscU/HrpY family**	1770922	1769840	Cellular processes; Protein fate
II	**BMA_A1632**	**type III secretion protein, HrpB1/HrpK family**	1771158	1771733	Protein fate
II	**BMA_A1633**	**type III secretion protein HrpB2**	1771747	1772163	Protein fate

II	**BMA_A1634**	**type III secretion inner membrane protein SctJ, authentic frameshift**	1772166	1773012	Cellular processes; Protein fate
II	**BMA_A1635**	**type III secretion protein HrpB4**	1773009	1773686	Protein fate
II	**BMA_A1636**	**type III secretion inner membrane protein SctL**	1773671	1774387	Cellular processes; Protein fate
II	**BMA_A1637**	**type III secretion apparatus H+-transporting two-sector ATPase**	1774423	1775712	Cellular processes; Protein fate
II	BMA_A1641	conserved hypothetical protein	1777605	1779371	Hypothetical proteins
II	BMA_A1640	hypothetical protein	1777625	1777221	
II	BMA_A1642	putative syringomycin biosynthesis enzyme	1780569	1779616	Cellular processes
II	BMA_A1643	non-ribosomal peptide synthase	1790695	1780637	Cellular processes
